# Sanguinarine triggers intrinsic apoptosis to suppress colorectal cancer growth through disassociation between STRAP and MELK

**DOI:** 10.1186/s12885-018-4463-x

**Published:** 2018-05-21

**Authors:** Xianling Gong, Zhihong Chen, Qinrui Han, Chunhui Chen, Linlin Jing, Yawei Liu, Liang Zhao, Xueqing Yao, Xuegang Sun

**Affiliations:** 1 The key laboratory of molecular biology, State Administration of Traditional Chinese Medicine, School of Traditional Chinese Medicine, Southern Medical University, Guangdong, Guangzhou, (510515) China; 2 School of pharmacy, Guangdong Medical University, Guangdong, Dongguan, (523808) China; 3 Traditional Chinese Medicine Integrated Hospital, Southern Medical University, Guangdong, Guangzhou, (510315) China; 4 Nanfang hospital, Southern Medical University, Guangzhou, (510515) China; 5 Department of Gastrointestinal Surgery, Guangdong General Hospital, Guangzhou, (510120) China

**Keywords:** Colorectal cancer, Sanguinarine, MELK, STRAP, Intrinsic apoptosis, Bax

## Abstract

**Background:**

Previous studies showed sanguinarine induced apoptosis in CRC cells but did not define the underlying mechanisms. The purpose of this work was to determine the in vivo and in vitro effects of sanguinarine on CRC tumors and to elucidate the mechanism in regulating the intrinsic apoptosis.

**Methods:**

Cell viability of CRC cell lines treated with sanguinarine was measured by MTT assay. Apoptotic cells stained with Annexin V and 7-AAD were detected by flow cytometry. Mitochondrial membrane potential and reactive oxygen species (ROS) were analyzed by JC-1 and DCFH-DA staining, respectively. The in vitro kinase activity of MELK was analyzed by using HTRF^®^ KinEASE™-STK kit. The expression of proteins were determined using Western blotting and immunohistochemistry. Co-immunoprecipitation and immunofluorecence were used to study the interaction between STRAP and MELK. The anti-neoplastic effect of sanguinarine was observed in vivo in an orthotopic CRC model.

**Results:**

Sanguinarine decreased the tumor size in a dose-dependent manner in orthotopical colorectal carcinomas through intrinsic apoptosis pathway in BALB/c-nu mice. It significantly increased cleavage of caspase 3 and PARP in implanted colorectal tissues. Sanguinarine increased mitochondrial ROS and triggered mitochondrial outer membrane permeabilization in multiple colorectal cancer (CRC) cell lines. NAC pretreatment lowered ROS level and downregulated apoptosis induced by sanguinarine. The intrinsic apoptosis induced by sanguinarine was Bax-dependent. The elevated expression and association between serine-threonine kinase receptor-associated protein (STRAP) and maternal embryonic leucine zipper kinase (MELK) were observed in Bax positive cells but not in Bax negative cells. Sanguinarine dephosphorylated STRAP and MELK and disrupted the association between them in HCT116 and SW480 cells. The expression and association between STRAP and MELK were also attenuated by sanguinarine in the tumor tissues. Importantly, we found that STRAP and MELK were overexpressed and highly phosphorylated in colorectal adenocarcinomas and their expression were significantly correlated with tumor stages. Furthermore, the expression of MELK, but not STRAP, was associated with lymph node metastasis.

**Conclusions:**

Sanguinarine dephosphorelates STRAP and MELK and disassociates the interaction between them to trigger intrinsic apoptosis. Overexpression of STRAP and MELK may be markers of CRC and their disassociation may be a determinant of therapeutic efficacy.

**Electronic supplementary material:**

The online version of this article (10.1186/s12885-018-4463-x) contains supplementary material, which is available to authorized users.

## Background

Colorectal cancer (CRC) is the third most common cancers expected to occur and the third most common cause of cancer deaths both in men and women in the United States in 2017 [1]. Even multiple modules of treatment including surgical resection and systemic infusional chemotherapy have been applied, the overall 5 year death rate for CRC patients is still about 34% [2].

Apoptosis includes two main pathways which are termed “extrinsic pathway” and “intrinsic pathway” that involve cell surface death receptors or the mitochondria respectively [3]. The pro-apoptotic proteins Bax or Bak of Bcl-2 family constitute the central effector of the intrinsic pathway [4]. Mitochondria have key roles in intrinsic apoptosis execution. Mitochondrial reactive oxygen species (ROS) signaling increases longevity through the intrinsic apoptosis pathway in the nematode *C. elegans* [5]. Chemotherapy promotes survival in CRC patients through ROS mediated apoptosis [6, 7]. Bax is a key regulatory role in inducing the mitochondrial outer membrane permeabilization (MOMP) [8]. Mitochondria, the convergence of pro-apoptotic proteins and redox, orchestrate the sequential events of MOMP and intrinsic apoptosis.

Serine-threonine kinase receptor-associated protein (STRAP) is a TGF-β receptor-interacting protein that participates in the regulation of cell proliferation and cell death [9]. Maternal embryonic leucine zipper kinase (MELK) is a member of the AMP-activated protein kinase-related kinase family and controls a variety of biological processes, including cell cycle, cell proliferation, carcinogenesis, and apoptosis [10]. STRAP is a positive regulators of MELK and MELK phosphorylates STRAP at Ser188 via direct interaction [9]. Althrough STRAP [11, 12] and MELK [10, 13–15] are highly expressed in multiple human cancers, their association in tumor progression and as a therapeutic target is largely unknown.

*Macleaya cordata* is a kind of commonly used traditional medicinal plants that first described in Ben Cao Shi Yi (“Supplement to the Materia Medica”) in Tang dynasty [16]. Sanguinarine is a major bio-active component that belongs to benzylisoquinoline alkaloid from *Macleaya cordata*. It has been demonstrated that sanguinarine could induce apoptosis in head and neck cancer cells [17], human colorectal cancer [18] and drug-resistant non-small-cell lung cancer cells [19]. Sanguinarine increased the generation of reactive oxygen species (ROS) and activation of c-Jun-N-terminal kinase (JNK) and nuclear factor-kappa B (NF-kappa B) [17]. We suppose that sanguinarine dictates the apoptosis-related collapse of the mitochondrial membrane potential to suppress the growth of CRC [20]. The role of STRAP and MELK in regulating MOMP induced by sanguinarine would be documented in our research.

## Methods

### Regeants

Antibodies against Caspase-3 (No. 9662), PARP (No. 9532), Pro-caspase 9 (No. 9508), Cleaved Caspase-9 (No. 7237), Cytochrome C (No. 4272), Bcl-2 (No. 2870), Bax (No. 5023) and PCNA (No. 13110) were from Cell Signaling Technology (CST), USA. Anti-maternal embryonic leucine zipper kinase (MELK) (No. ab155767) was obtained from Abcam, UK. Anti-serine-threonine kinase receptor-associated protein (STRAP) (No. sc-377,345) and Caspase 8 (No. sc-56,070) antibody was purchased from Santa Cruz Biotechnology, USA. Anti-phospho-MELK (Thr167, Ser171) (No. WG-00203P) and Anti-phospho- STRAP (Thr175, Ser179) (No. WG-00204P) were ordered from ABclonal Technology, China. Alexa fluor^®^ 488 goat anti-rabbit IgG(H + L) (No. CA11008s) and Alexa fluor^®^546 goat anti-mouse IgG(H + L) (No. A11003) were obtained from Molecular Probes (Invitrogen). Beta-actin antibody (No. E021020–01) was from EarthOx, LLC, San Francisco, USA. Sanguinarine was purchased from National Institutes of Food and Drug Control (Beijing, China). Sanguinarine was dissolved in DMSO (MP, France) and diluted in culture medium for each experiment. The final concentration of DMSO didn’t exceed 0.1%.

### Cell lines and cell culture

Human colon carcinoma cell lines SW480 (cat. No. TCHu172) and HCT116 (WT) (cat. No. TCHu 99) were purchased in 2014 from the Chinese Academy of Science Committee Type Culture Collection Cell Bank (Shanghai, China). HCT116 *Bax*^*−/−*^ human colon carcinoma cell line was a generous gift from Dr. Bert Vogelstein (Howard Hughes Medical Institute, MD). SW480 and HCT116 (WT) cells were cultured in RPMI 1640 medium (Gibco) and HCT116 *Bax*^*−/−*^ cells in McCoy’s 5A medium (Invitrogen). The medium was supplemented with 10% fetal bovine serum (Gibco) and 1% penicillin-streptomycin (Invitrogen). All cells were maintained at 37 °C in a humidified incubator of 5% CO_2_-containing atmosphere.

### Cell viability assay

Cells were seeded in 96 wells plates at density of 0.8 × 10^4^ cells per well. Sanguinarine was dissolved in DMSO as a stock solution and was stored in aliquots at − 20 °C. After treatment with or without sanguinarine, MTT (3-[4, 5-dimethylthiazolyl-2]-2, 5-diphenyl tetrazolium bromide) (MP, France) was added into each well. Cells were incubated for 4 h. The formazan crystals were dissolved in DMSO. The absorbance value was measured at 490 nm.

### Hoechst 33,342 staining

Hoechst 33,342 (MP, France) staining was performed according to the manufacturer’s instructions. Morphological changes in nuclei were observed under a fluorescence microscope using a blue filter (NIKON ECLIPSE T*i*-S).

### PE-Annexin V/7-AAD staining assay

Cells were stained with PE-Annexin V/7-AAD (BD Bioscience pharmingen) according to the manufacturer’s instructions. Cells were resuspended in 500 μL 1 x binding buffer, 5 μl PE-Annexin V and 5 μl 7-AAD were added to the sample and incubated at room temperature for 15 min in the dark. The stained samples were then detected by flow cytometry (BD FACSCalibur).

### Western blotting

Total cells were lysed in RIPA buffer. Mitochondrial and cytoplasmic fractions were isolated according to the manufacturer’s instructions of Cell Mitochondria Isolation Kit (Beyotime, China). Mitochondria were lysed in RIPA buffer. The protein concentrations of the mitochondria, cytoplasm and whole cells were determined by the BCA (Thermo scientific) method using the Thermo protein assay kit. Equal amount of proteins from each group were subjected to SDS-PAGE on 12% gel, transferred to a PVDF membrane (Millipore) by electroblotting, blocked with 5% nonfat milk for 1 h at room temperature and incubated with primary antibodies overnight at 4 °C. Next, the membrane was incubated by HRP-conjugated appropriate secondary antibodies, visualized by enhanced chemiluminescence (Millipore) and exposed by KODAK Image Station 4000MM Digital Imaging System [21, 22].

### JC-1 staining method

Cells were stained with JC-1 according to the manufacturer’s instructions of Mitochondrial membrane potential assay kit (Beyotime, China). Cells were then detected using flow cytometry (BD FACSCalibur). The mitochondrial membrane potential was calculated based on the following equation: mitochondrial membrane potential = red fluorescence intensity / green fluorescence intensity.

### Detection of ROS generation

After treatment with sanguinarine of different concentration for indicated time, cells were stained with 5-(and 6)-carboxy-2′,7′-dichlorodihydrofluorescein diacetate (DCFH-DA) according to the manufacturer’s instructions of Reactive Oxygen Species Assay Kit (Beyotime, China). Intracellular production of ROS was evaluated by flow cytometry. Relative Ros levels = DCH fluorescence intensity(treatment group) / DCH fluorescence intensity (control group).

### QRT-PCR

Total RNA was extracted with TRIzol (Takara, Dalian, China). 2 μg of the total RNA was reversely transcribed using a reverse transcription kit (Takara) according to the manufacturer’s protocols and cDNA was obtained. The primers for STRAP were 5’-AAGGGACACTTTGGTCCTATTC-3′ (fwd), 5’-CCTACCACAGTTTGCCATAGT-3′ (rev). The primers for MELK were 5′- ACTTGCCTGCCATATCCTTAC-3′ (fwd), 5’-GGTTCTTCAAGGCCTCAATCT -3′ (rev).The primers for GAPDH were 5’-ATTGTCAGCAATGCATCCTG-3′ (fwd), and 5′-ATGGACTGTGGTCATGAGCC-3′ (rev). All reactions were performed in triplicate for 40 cycles in a Stratagene Mx3005P system as previously described [23]. Relative expression was calculated with GAPDH using the 2^-ΔΔCt^.

### Immunoprecipitation

Immunoprecipitation was performed as previously described [24]. Briefly, cell extract was mixed with anti-STRAP, anti-MELK antibody at 4 °C overnight. Agarose beads were added at a ratio of 1 mg of extract per 120 μl of agarose at 4 °C for 3 h. The beads were then pelleted at 2500×g for 3 min and washed with lysis buffer five times. The beads were subjected to elution with 5 vol of 0.5 mg/ml peptide for 4 h or directly boiled in loading buffer.

### Immunofluorescence

Cells were fixing with 4% paraformaldehyde at room temperature for 25 min and permeabilized using 0.2% Triton-100X and then blocked by 5% BSA at room temperature for 1 h. Next, they were incubated with primary antibody overnight at 4 °C according to the manufacturer’s instructions. Then, the cells were followed by staining with secondary antibodies (Alexa Fluor 488 and 546, Invitrogen). Cell nuclei were stained with DAPI at 37 °C in darkness for 8 min. Pictures were taken under confocal microscope (NIKON C2^+^).

### Kinase activity assay

The in vitro kinase activity of MELK was analyzed by using HTRF^®^ KinEASE™-STK kit. It is a generic method for measuring Serine/Treonine kinase activities. The assay was performed according to manufacturer’s protocol. MELK Kinase was incubated with sanguinarine and the STK Substrate-biotin for 50 min at room temperature in the presence of ultrapure ATP. STK Antibody labeled with Eu3^+^-Cryptate STK Substrate-biotin and streptavidin-XL665 were added to the mixture in a single addition with EDTA. They were incubated for 60 min at room temperature to stop the kinase activity. The kinase activity of MELK were detected by Tecan Infinite® 200 PRO microplate spectrophotometer.

### Orthotopic CRC model

6 to 8 week-old BALB/c-nu male nude mice (NO. 44002100007706) were purchased from Experimental Animal Centre of Southern Medical University. The mice were housed in a specific pathogen-free facility and maintained under 12 h light/dark cycles. All animal studies were performed according to protocols of the Care and Use of Laboratory Animals published by the National Institutes of Health and approved by the Laboratory Animals Care and Use Committee of Southern Medical University. Orthotopic CRC model on nude mice was established according to our previously described methods [24]. Twenty-Four tumor-bearing male nude mice were randomly divided into four groups (*n* = 6 each group) treated with (1) Control diluents; (2) sanguinarine 4 mg/kg/d by administeration via oral gavage; (3) sanguinarine 8 mg/kg/d by administeration via oral gavage; (4) cisplatin 1 mg/kg/d by intraperitoneal injection for 21 days**.** Nude mice were weighted every 5 days. The mice were anesthetized with Sodium Pentobarbital 100 mg/kg and then sacrificed at the end of the experiment according to the euthanasia guidelines for experimental animals of Southern Medical University. The tumor tissues were dissected and fixed in 4% paraformaldehyde for histologic examination or stored at − 80 °C for western blotting.

### Tumor tissue samples

The collections of tumor and adjacent tissue samples were approved by the Ethics Committee of Guangdong General Hospital and all aspects of the study comply with the Declaration of Helsinki. Informed consents were signed voluntarily by all patients that included in this study. Fresh primary CRC specimens and paired noncancerous colorectal tissue were provided by the Tumor Tissue Bank of Guangdong General Hospital. In each case, a diagnosis of primary CRC had been made, and the patient had undergone elective surgery for CRC in Guangdong General Hospital between 2015 and 2016. The pathological diagnosis was made in the Department of Pathology of Guangdong General Hospital.

### Immunohistochemistry

Tumour tissues from control and treated mice were obtained after sacrificing the mice and then fixed in 4% paraformaldehyde and embedded in paraffin. Sections were deparaffinized, rehydrated. Endogenous peroxidase activity was blocked with 3% (*v*/v) hydrogen peroxide solution. Heat-induced antigen retrieval was performed. Sections were incubated with 5% BSA to block no-specific staining. After incubation with the primary antibodies (anti-MELK, anti-STRAP, anti-Bax) overnight at 4 °C in a humid chamber, sections were incubated with mouse/rabbit-labelled polymer from GTVision™ III kit (Gene Tech, China) for 1 h at 37°C. Positive signals were visualized by DAB kit. The slides were reviewed by two or three pathologists blind to the study. To evaluate the expression levels, immunostained slides were evaluated using a method described previously [25]. TUNEL assay was performed according to manufacturer’s protocol of In Situ Cell Death Detction Kit, TMR red (Roche, Mannhem, Germany).

### Statistical analysis

Parallel experiments were repeated three times. Data was analyzed using SPSS 22.0 statistical software. And results are expressed as: mean ± standard deviation (SD). Mean was compared between groups using one Way-ANOVA. When the variance was homogeneous, *LSD* was used for multiple comparisons. *Dunnett T3* method was applied for the unequal variances. Mean of two groups was compared with independent samples t test. A value of *p* < 0.05 indicated statistically significant difference.

## Results

### Sanguinarine inhibits the growth of orthotopical implanted CRC by inducing apoptosis

**Fig. 1:**
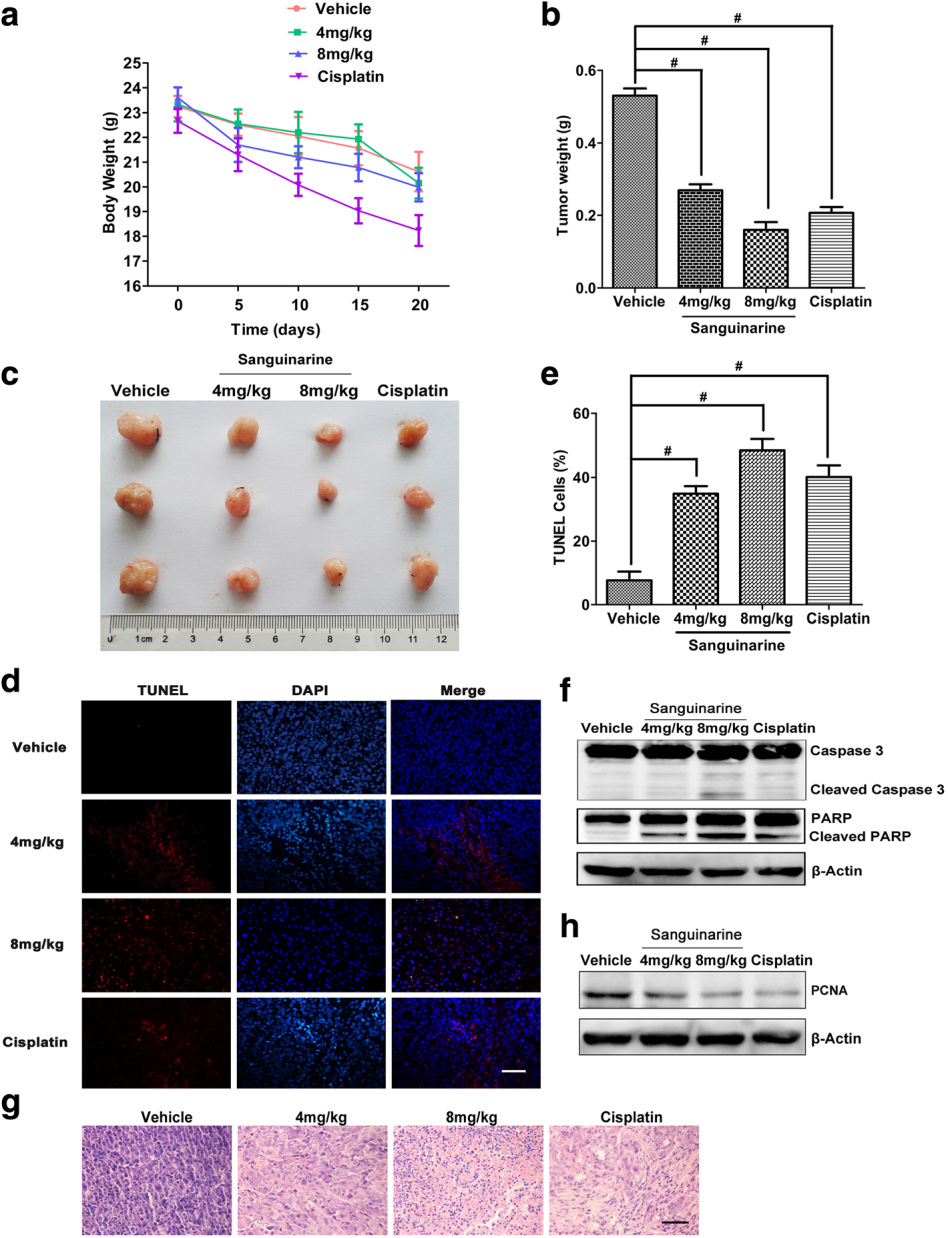
Sanguinarine inhibited the growth of orthotopic CRC in nude mice by inducing apoptosis. **a** Effect of sanguinarine and Cisplatin on body weight of nude mice bearing xenografts. **b** Photographs of tumors were from four groups. **c** Tumor weight of mice from Orthotopic implanted CRC model. **d** Tumors were dissected and tested using TUNEL staining kit (red) and DAPI (blue). The slides were observed by inverted fluorescence microscope (400×) (Scale bar is 50 μm). **e** Apoptosis was analyzed quantitatively by calculating percentage of the number of cells with TUNEL staining as compared to the total number of cells. **f** The apoptosis corresponding proteins from tumor tissue were analyzed by western blotting. **g** Representative photographs of H&E staining (400×) (Scale bar is 50 μm). **h** PCNA protein from tumor tissue was detected by western blotting. Data presented are showed as means ± SD from three independent experiments. ^#^ *p* < 0.01 indicates significant difference

An orthotopically implanted CRC model was used to characterize the anti-tumoral effects of sanguinarine in vivo [24]. The tumor weight was significantly reduced by both sanguinanine and Cisplatin in situ significantly (Fig. 1b and c). However, the body weight loss caused by sanguinarine was lower than that of caused by cisplatin (Fig. 1a). Increased TUNEL positive cells indicate that the cell death induced by sanguinarine can be defined as apoptosis (Fig. 1d and e). Western blot analysis showed that cleaved PARP and caspase 3 were increased significantly in sanguinarine-treated tissues (Fig. 1f). Further evidence indicated that cell proliferation was dampened by sanguinarine treatment (Fig. 1g and h). These results suggested that the growth of orthotopical implanted CRC was suppressed by sanguinarine via the induction of apoptosis.

### Sanguinarine induces intrinsic apoptosis in CRC cell lines

**Fig. 2:**
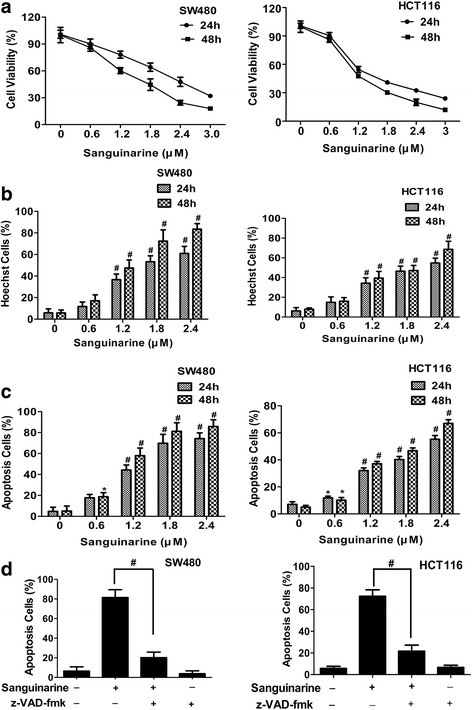
Sanguinarine-induced apoptosis in CRC cells. **a** SW480 and HCT116 cells were treated with the indicated concentrations of sanguinarine for 24 h and 48 h. Cell viability was measured by MTT assay (*n* = 5). **b** Cells were stained by DNA-specific Hoechst 33,342 dye. Hoechst positive cells were analyzed quantitatively by calculating the percentage of the number of cells with condensed chromatin (*n* = 3). **c** Cells were stained with PE-Annexin V and 7-AAD and quantitatively analyzed by flow cytometry (*n* = 3). **d** Cells were pretreated with or without pan-caspase inhibitor, z-VAD-fmk (50 μM) respectively, for 1 h before explosure to sanguinarine for 48 h. Apoptosis cells were evaluated as described in (**c**) (*n* = 3). Data presented are showed as means ± SD from three independent experiments.* *p* < 0.05 and ^#^ *p* < 0.01 indicate significant difference

Sanguinarine decreased the cell viability in dose- and time- dependent manner both in SW480 and HCT116 colorectal adenocarcinoma cells as monitored by 3-[4,5-dimethylthiazolyl-2]-2,5-diphenyl tetrazolium bromide staining at 24 h and 48 h (Fig. 2a). The number of Hoechst positive cells increased simultaneously upon treatment of sanguinanine (Fig. 2b). Chromatin condensation, rounding-up of the cells, cell shrinkage and extensive detachment of the cells from the cell culture substratum were observed by Hoechst 33,342 staining (Additional file 1: Figure S1 and Additional file 2: Figure S2). Flow cytometry showed that annexin V positive cells were significantly increased by sanguinarine treatment at 24 h and 48 h in a dose-dependent manner (Fig. 2c). Analysis of flow cytometry were presented in Additional file 3: Figure S3. To further define the cell death subroutines, cells were preincubated with a pan-caspase inhibitor, N-benzyloxycarbonyl-Val-Ala- Asp(OMe)-fluoromethyl ketone (Z-VAD-FMK). The annexin V positive cells were significantly reduced and the viability were elevated correspondingly by Z-VAD-FMK pretreatment (Fig. 2d and Additional file 4: Figure S4). These results suggested that sanguinarine induced apoptosis is the main cause for the reduced cell viability.

### Sanguinarine increase ROS to induce MOMP

**Fig. 3:**
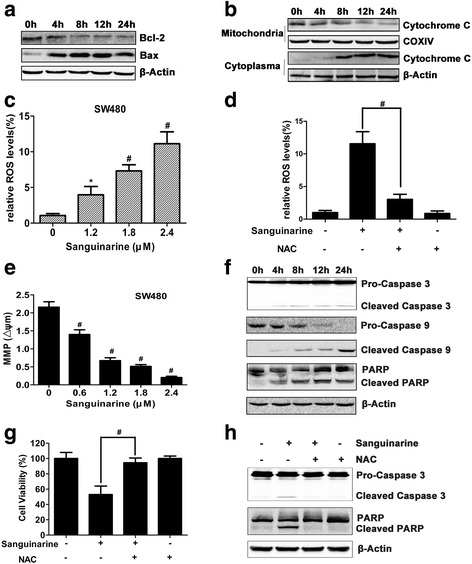
Sanguinarine-induced apoptosis of CRC cells is an intrinsic apoptosis. **a** The expression of proteins from SW480 cells treated with 2.4 μM sanguinarine of Bcl-2 and Bax were determined by Western blotting. **b** Change of cytochrome c in mitochondrial and cytoplasmic fractions was analyzed by western blotting. COXIV and β-Actin were used to confirm equal protein loading respectively. **c** Cells were treated with the indicated concentrations of sanguinarine. ROS levels were evaluated by flow cytometry. **d** Cells were treated with or without 2.4 μM sanguinarine for 12 h after pretreatment with or without 10 mM NAC, a ROS scavenger, for 1 h. ROS levels were evaluated by flow cytometry. **e** Mitochondria membrane potential (ΔΨm) of SW480 cells was evaluated by JC-1 ratio of red and green fluorescence after sanguinarine treatment for 48 h. **f** Whole cell lysates were subject to western-blotting analysis using an antibody against caspase-3, caspase-9, cleaved caspase-9 and PARP antibodies. **g** Cell viability was measured by MTT assay (n = 5). **h** Expression of the apoptosis proteins from SW480 cells treated with or without 2.4 μM sanguinarine after pretreatment with or without 10 mM NAC for 1 h. Data presented are showed as means ± SD from three independent experiments. * *p* < 0.05 and ^#^ *p* < 0.01 indicate significant difference

Apoptosis is a highly coordinated process that orchestrates a series of biochemical events. It recruits the pro-apoptotic members of the Bcl-2 family, such as Bax or Bak to control the formation of the mitochondrial apoptosis-induced channel (MAC) [26]. Sanguinarine induced the activation of Bax and downregulated the expression of Bcl-2 (Fig. 3a). The activation of Bax was accompanied by the release of cytochrome *c* from mitochondria to cytoplasm (Fig. 3b). The translocation of cytochrome *c* suggested the opening of MAC and mitochondrial outer membrane permeabilization (MOMP). DCFH-DA staining further showed that the ROS was significantly elevated by sanguirnarine, which can be effectively suppressed by L-N-acetyl-cysteine treatment (Fig. 3c and d).

JC-1 staining showed that the mitochondrial membrane potential (MMP) was significantly reduced by sanguinarine in SW480 cells (Fig. 3e and Additional file 5: Figure S5). In the cytosol, cytochrome *c* binds to Apaf-1, thereby triggering hierarchical activation of caspase-9 and caspase-3 [27]. The cleavage of caspase-9 and caspase-3, and subsequent cleavage of PARP were observed in SW480 cells treated with sanguinarine (Fig. 3f). The cell viability was restored by NAC pretreatment and the cleavage of caspase-3 and PARP was downregulated accordingly in cells treated with sanguinarine (Fig. 3g and h). Taken together, these results suggested that sanguinarine induces intrinsic apoptosis by triggering MOMP [24, 28].

### Sanguinarine induces intrinsic apoptosis in a Bax-dependent manner

**Fig. 4:**
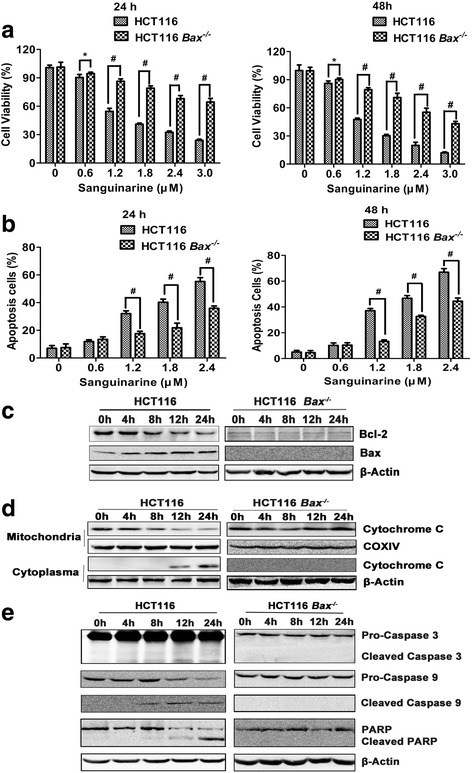
Sanguinarine-induced apoptosis of CRC cells was Bax-depedent. **a** HCT116 WT and HCT116 *Bax*^*−/−*^ cells were incubated with various concentrations of sanguinarine for 24 h and 48 h. Cell viability was detected by MTT assay (*n* = 5). **b** Apoptosis cells were quantifiedby flow cytometry after PE-Annexin V and 7-AAD staining (*n* = 3). **c** Whole lysates were subjected to western blotting for expression of proteins of Bcl-2 and Bax. **d** Immunoblot was performed to evaluate cytochrome C in mitochondrial and cytoplasmic fractions. COXIV and β-Actin were served as loading control respectively. **e** The apoptosis regulatory proteins of whole lysates were analyzed by western blotting. Data presented are showed as means ± SD from three independent experiments. * *p* < 0.05 and ^#^ *p* < 0.01 indicate significant difference

To determine if the sanguinarine induced apoptosis is Bax-dependent, HCT116 *Bax*^*+/+*^ (wild type) and HCT116 *Bax*^*−/−*^ cells were adopted [24]. Our results showed that the reduced cell viability by sanguinarine was significantly antagonized in HCT116 *Bax*^*−/−*^ cells (Fig. 4a). Correspondingly, sanguinarine-induced apoptosis was significantly lowered in HCT116 *Bax*^*−/−*^ cells (Fig. 4b). The apoptosis induced by sanguinarine in HCT116 *Bax*^*−/−*^ cells was partly rescued by Bax transfection. But it wasn’t rescued by transfection of Bak (Additional file 6: Figure S6). The activation of Bax and downregulation of Bcl-2 were both abolished in HCT116 *Bax*^*−/−*^ cells (Fig. 4c). JC-1 staining further confirmed that for HCT116 (WT), the mitochondrial membrane potential decreased more significantly than that of HCT116 *Bax*^*−/−*^ (Additional file 7: Figure S7). Accordingly, the release of cytochrome *c* from mitochondria (Fig. 4d) and the cleavage of caspase-9, and casapase-3 were near totally abrogated in HCT116 *Bax*^*−/−*^ cells (Fig. 4e), either. These results provided consolidate evidence that Bax is a key regulator in sanguinarine-induced apoptosis [29].

### Sanguinarine reduces the expression of STRAP and MELK

**Fig. 5:**
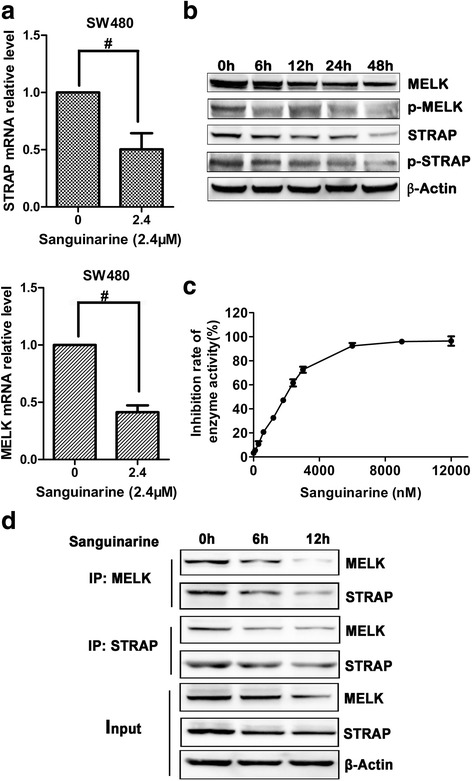
Sanguinarine induced apoptosis of CRC cells by inhibition of the interaction of MELK and STRAP. **a** SW480 cells were treated with sanguinarine for 12 h. STRAP and MELK mRNA relative level was detected by qRT-PCR. **b** Western blotting analysis of MELK, STRAP, p-MELK and p-STRAP in SW480 cells after treatment of 2.4 μM sanguinarine for specified times. **c** The in vitro kinase activity of MELK was analyzed by using HTRF^®^ KinEASE™-STK kit. **d** The interaction between MELK and STRAP was analyzed by Co-IP. Cells were lysed and immunoprecipitated using an anti-MELK or STRAP antibody. The precipitated proteins were analyzed using westernblotting. Data presented are showed as means ± SD from three independent experiments. ^#^ *p* < 0.01 indicates significant difference

STRAP is up-regulated in 60% colon carcinomas and demonstrates oncogenic function in multiple tumors [11]. qRT-PCR (Fig. 5a) and western blot (Fig. 5b) were used to observe the effects of sanguinarine on the expression of STRAP. These results showed that sanguinarine significantly reduced its expression.

It has been previously reported that STRAP was a medium of bonding between maternal embryonic leucine zipper kinase (MELK) and TGF-β to regulate cell proliferation and apoptosis [9]. Interestingly, sanguinarine also decreased the expression of MELK in a time-dependent manner (Fig. 5b). Furthermore, the phosphorylation of STRAP and MELK was downregulated in a dose dependent manner by sanguinarine treatment (Fig. 5b). In vitro kinase assay showed that the kinase activity of MELK was significantly downregulated by sanguinarine in a dose-dependent manner (Fig. 5c). Our experiments showed that interaction between STRAP and MELK was significantly disrupted in SW480 cells upon sanguinarine treatment (Fig. 5d). These data suggested that sanguinarine lowers the expression and phosphorylation of STRAP and MELK and disassociates the interaction between them.

### Sanguinarine disassociates STRAP and MELK to induce Bax-dependent apoptosis

**Fig. 6:**
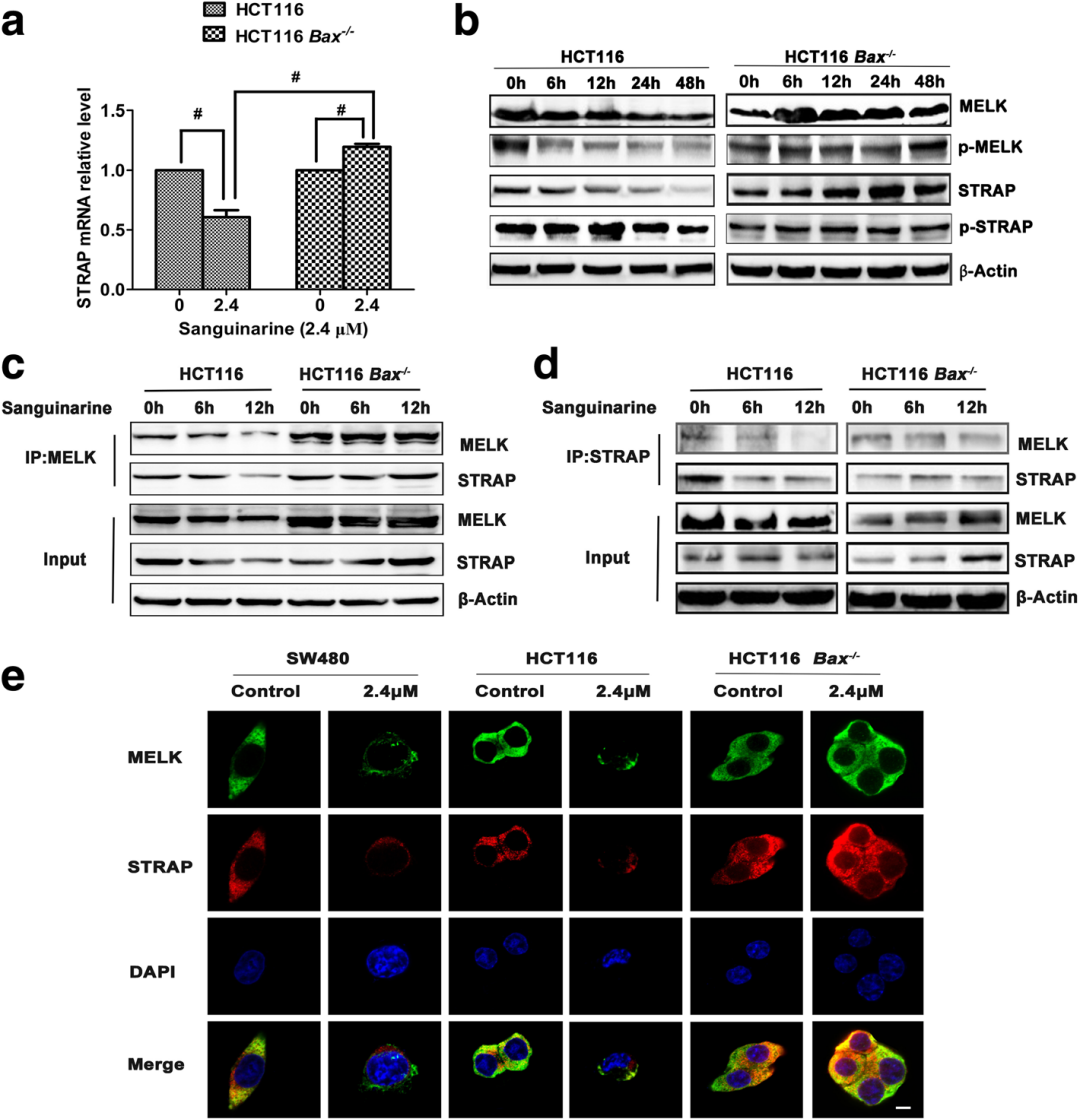
Sanguinarine induces Bax-dependent disassociation between STRAP and MELK. **a** HCT116 WT and HCT116 *Bax*^*−/−*^ cells were treated with sanguinarine for 12 h. RNA was extracted and qRT-PCR were performed to analyze STRAP mRNA relative level. **b** Protein expression of MELK, STRAP, p-MELK and p-STRAP was detected by western blotting. **c** and **d** The interaction between MELK and STRAP was analyzed by Co-IP. The lysate was subjected to immunoprecipitation with MELK **(c)** and STRAP **(d**) antibody respectively. Interaction of MELK and STRAP was evaluated by western blotting. **e** SW480, HCT116 WT and HCT116 *Bax*^*−/−*^ cells were treated respectivly with sanguinarine for 12 h and fixed by 4% paraformaldehyde. Cells were incubated with antibodies respectively and then probed secondary antibodies conjugated with FITC. Immunofluorescence imagines were observed under confocal microscopy (1000×) (Scale bar is 10 μm). Data presented are showed as means ± SD from three independent experiments. ^#^ *p* < 0.01 indicates significant difference

In breast cancer cells, MELK suppressed long isoform of Bcl-G (Bcl-G_L_)-induced apoptosis [30]. To verify the role of MELK in Bax-mediated apoptosis, its expressions in HCT116 and HCT116 *Bax*^*−/−*^ cells treated with sanguinarine were documented. Contrary to the significantly reduced expression in sanguinarine treated HCT116, the expression of MELK and STRAP in HCT116 *Bax*^*−/−*^ cells was slightly elevated (Fig. 6a and Additional file 8: Figure S8). Western blot provide further evidence that sanguinarine decreased the phosphorylation and expression of STRAP and MELK in HCT116 but increased their expression slightly in HCT116 *Bax*^*−/−*^ cells at the same time (Fig. 6b). To test if the specific association between STRAP and MELK was regulated by Bax, we probed the immunocomplex using anti-STRAP and anti-MELK antibody to immunoprecipitate each other in HCT116 and HCT116 *Bax*^*−/−*^ cells, respectively. As expected, the interaction between STRAP and MELK was weakened by sanguinarine in HCT116 but was enhanced in HCT116 *Bax*^*−/−*^ cells (Fig. 6c and d). Immunofluorescence confirmed the disassociation between STRAP and MELK in Bax positive cell treated with sanguinarine while showed strengthened association in HCT116 *Bax*^*−/−*^ cells (Fig. 6e). These results suggested that the disrupted association between STRAP and MELK by sanguinarine was Bax-dependent.

### Sanguinarine induces apoptosis in vivo through downregulation and disassociation of STRAP and MELK

**Fig. 7:**
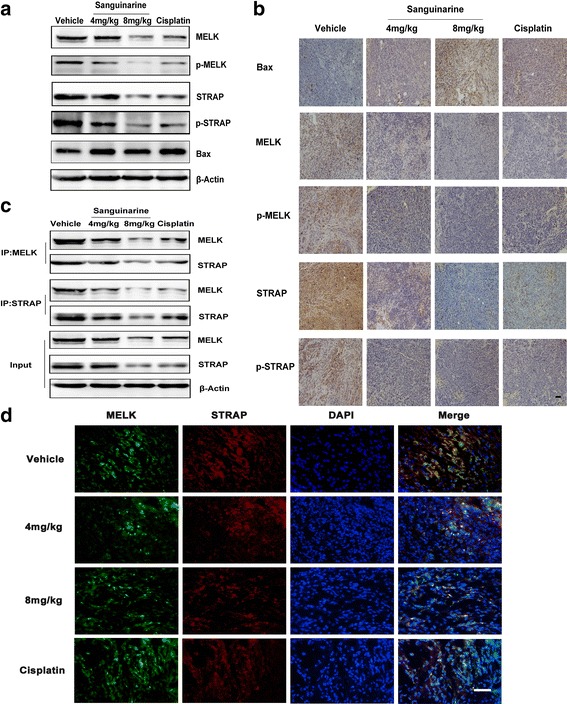
Sanguinarine induces apoptosis in vivo through disassociation of STRAP and MELK. **a** The expression of MELK, p-MELK, STRAP and p-STRAP in tumor tissue was detected by western blotting analysis. **b** Representative images of immunohistochemical staining of Bax, MELK p-MELK, STRAP and p-STRAP in tumor tissues (200×) (Scale bar is 50 μm). **c** Primary antibodies of MELK and STRAP were added into the whole lysates from tumor tissue respectively. Co-IP MELK and STRAP was tested by western blotting. **d** Tumors from four groups were dissected, fixed and embedded using O.C.T gum. Interaction with STRAP and MELK was tested by double immune-fluorescence staining for MELK (green) and STRAP (red) in colon cancer tissues. Photoes were taken by inverted fluorescence microscope (400×) (Scale bar is 50 μm)

The expression of Bax was elevated and the expressions of STRAP and MELK were decreased by sanguinarine treatment (Fig. 7a and b). When baited with MELK, immunoprecipitated STRAP were significantly reduced both in sanguinarine and cisplatin treated tumor tissues extracts (Fig. 7c). When probed with STRAP, the attenuated interaction between STRAP and MELK was also observed. The interaction between MELK and STRAP was also suppressed by cisplatin (Fig. 7c). Immunofluorescence test showed that the association between MELK and STRAP was weakened significantly by sanguinarine (Fig. 7d). Collectively, our in vivo experiments again confirmed that sanguinarine decreases the growth of CRC through downregulation and disassociation of STRAP and MELK.

### STRAP and MELK enhance oncogenic potential in colorectal specimens

**Fig. 8:**
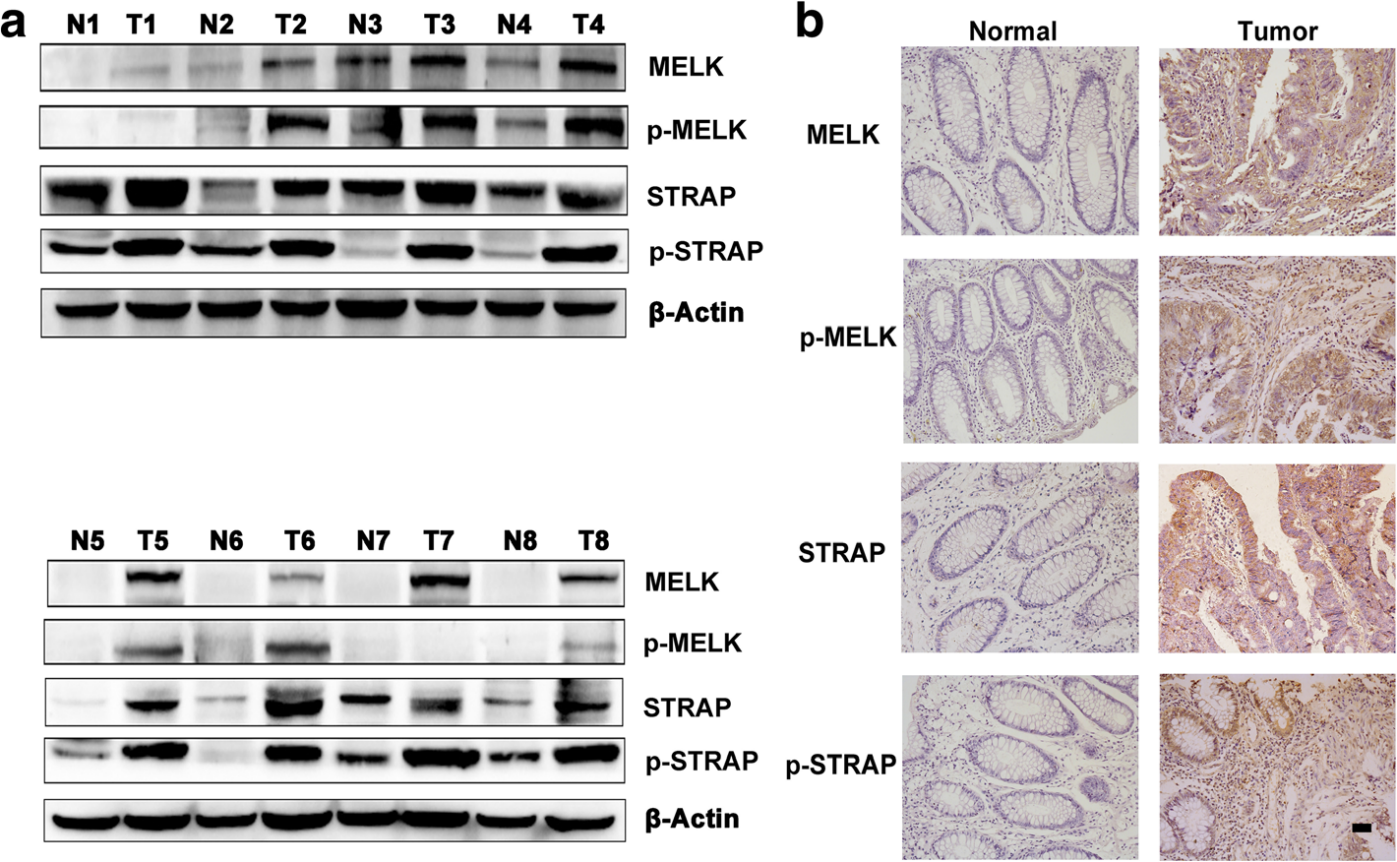
Elevated MELK, p-MELK, STRAP and p-STRAP expression in colorectal cancer. **a** Western blotting analysis of MELK, p-MELK, STRAP and p-STRAP in CRC tissues (T) and the patient-matched adjacent non-tumor tissues (N) was performed**.** β-Actin were used to confirm equal protein loading respectively. **b** Representative images of immunohistochemical staining of MELK, p-MELK, STRAP and p-STRAP in CRC tissues (T) and the patient-matched adjacent non-tumor tissues (N) (200×)(scale bar is 50 μm)

To further explore the role of STRAP and MELK in tumorigenesis of CRC, the phosphorylation and expression of them were checked in CRC and paired normal tumor-adjacent colorectal tissues. No significant correlation was found between the expression level of MELK, STRAP and patients’ age, gender, and tumor size. The expression and phosphorylation of MELK and STRAP were significantly elevated in CRC specimens as compared to the paired normal tissues (Fig. 8a and b). Both MELK and STRAP were significantly correlated with tumor stages. Furthermore, the expression of MELK, but not STRAP, was associated with lymph node metastasis (Table 1). These results suggested that both STRAP and MELK have oncogenic potential and might be promising molecular target for development of therapy for CRC patients.

**Table 1:** Correlation analysis of MELK, STRAP expression and clinical features

Characteristics	Patients *n* = 51	MELK expression			STRAP expression		
		low	high	*P* value	low	high	*P* value
Gender				0.972			0.945
Male	33	13	20		15	18	
Female	18	7	11		8	10	
Age (year)				0.389			0.741
≥ 60	32	14	18		15	17	
< 60	19	6	13		8	11	
Tumor size				0.431			0.461
≥ 5 cm	16	5	11		6	10	
< 5 cm	35	15	20		17	18	
Tumor differention				0.544			0.931
Good	0						
Moderate	47	19	28		21	26	
Poor	4	1	3		2	2	
T-stage				0.026			0.013
1–2	10	7	3		8	2	
3–4	41	13	28		15	26	
N-stage				0.024			0.991
0	31	16	15		14	17	
1–2	20	4	16		9	11	
M-stage				0.832			0.491
0	44	17	27		19	25	
1	7	3	4		4	3	

## Discussion

Our experimental data provide direct evidence that sanguinarine suppresses the growth of CRC through inducing intrinsic apoptosis in vivo. It induced Bax-dependent apoptosis in multiple CRC cell lines both in dose- and time-dependent manner. It is consistent with the reports that sanguinarine promotes apoptosis in human CRC, bladder cancer, oral squamous cell carcinoma and primary effusion lymphoma cell lines, et al. [31–34]. However, these studies simply broached the subject of the effect of sanguinarine on Bax expression but did not define the underlying mechanisms. Our current study focused on these unaddressed mechanisms and found that MOMP is activated by sanguinarine and Bax is a key regulator in sanguinarine induced MOMP. Cytochrome *c* is thus released from mitochondria into cytosol and subsequently triggers hierarchical activation of caspase-9, caspase-3 and caspase-7. Furthermore, we investigated that sanguinarine induced extrinsic apoptosis in CRC cells (Additional File 9: Figure S9).

Our results demonstrate that sanguinarine downregulates the expression of STRAP and MELK. Increased MELK expression has been identified in multiple human cancers: prostate [14], breast [13], brain [35, 36], colorectal [37], gastric [38] and lung cancer [15]. The high expression of MELK is correlated aggressive subtypes and poor prognosis of breast cancer [13] and poorly differentiated phenotypes in human astrocytoma [35] and prostate cancers [14]. Upregulation of MELK drives cell cycle progression and tumor formation and it has been shown to be differentially expressed in cancer stem cells or tumor-initiating cells [39]. Its expression localizes to colorectal carcinoma and in particular the basal regions of crypts of normal gastrointestinal epithelium where the location of stem cells in normal colonic tissue [37]. These data suggests that sanguinarine might suppress malignant transformation and proliferation of CRC through downregulating expression of MELK. It has been reported that MELK was associated with increased resistance of colorectal cancer cells against radiation and 5-FU [40], sanguinarine mightbe a candidate therapy for apoptosis resistant patients.

Serine-threonine kinase receptor-associated protein (STRAP) is a novel tryptophan-aspartate 40 (WD40) domain-containing protein [11]. It is an oncogenic protein that is up-regulated in 60% of colorectal cancers and 78% of lung cancers and increases proliferate potential of tumor cells [10, 11]. STRAP acts as a negative regulator of apoptosis signal-regulating kinase 1 and suppresses apoptosis in a dose-dependent manner [41]. It is consistent with our results that sanguinarine downregulates STRAP and induces apoptosis in CRC cell lines.

The orthotopical models showed the efficacy of sanguinarine with less body weight loss, suggested its translational potential as an anti-CRC agent with less toxicity. Sanguinarine downregulates the expression of MELK and STRAP in CRC tissues. Furthermore, it attenuates the association between MELK and STRAP. Sanguinarine induces intrinsic apoptosis in the presence of Bax. The weakened interaction of MELK and STRAP is necessary for the transactivation of Bax from cytosol to mitochondria. The accumulation of Bax in the mitochondria induces MOMP which cause the release of cytochrome *c* into the cytosol from mitochondria [24].

STRAP and MELK are highly expressed and phosphorylated in tissues of CRC patients. The strengthened association between them might be a marker for the formation of CRC. The attenuated formation of MELK and STRAP complex can be observed in cells treated with sanguinarine, and can also be induced by sanguinarine in mice bearing CRC tumors. These data provide evidence that sanguinarine induces intrinsic apoptosis through downregulation and disassociation of MELK and STRAP. MELK and STRAP are potential therapeutic targets that for triggering MOMP in CRC.

## Conclusions

STRAP and MELK are potential pro-tumoral markers of CRC. Sanguinarine shows efficacious anti-tumor effects through downregulating and dephosphorelating STRAP and MELK. Sanguinarine disassociates the interaction between STRAP and MELK to trigger intrinsic apoptosis.

## Additional files

Additional file 1: **Figure S1.** Apoptosis was detected in SW480 cells treated with sanguinarine. Cells were treated with the indicated concentrations of sanguinarine for 24 h and 48 h and then stained by DNA-specific Hoechst 33,342 dye. Imagines were observed by inverted fluorescence microscope (400×). Scale bar is 50 μm. (TIF 16868 kb)

Additional file 2: **Figure S2.** Apoptosis was detected in HCT116 cells treated with sanguinarine. Cells were treated with the indicated concentrations of sanguinarine for 24 h and 48 h and then stained by DNA-specific Hoechst 33,342 dye. Imagines were observed by inverted fluorescence microscope (400×). Scale bar is 50 μm. (TIF 16794 kb)

Additional file 3: **Figure S3.** Apoptotic cells were significantly increased by sanguinarine treatment. Indicated CRC cell lines were treated with the various concentrations of sanguinarine. To detect the degree of apoptosis, cells were analyzed by flow cytometry after PE-Annexin V and 7-AAD staining. (TIF 15845 kb)

Additional file 4: **Figure S4.** Z-VAD-fmk inhibited sanguinarine-induced apoptosis in CRC cells. After treatment with the combination of sanguinarine and Z-VAD-fmk (50 μM), cells were analyzed using flow cytometry. (TIF 5542 kb)

Additional file 5: **Figure S5.** Change of the mitochondrial membrane potential (MMP) in SW480 cells by sanguinarine. Cells were treated with the various concentrations of sanguinarine and stained with JC-1. Mean JC-1 fluorescence intensity was evalued by a flow cytometer. (TIF 2721 kb)

Additional file 6: **Figure S6.** Detection of Apoptosis in Bax-overexpressing or BAK-overexpressing HCT116 *Bax*^*−/−*^ cells treated with sanguinarine. **a** and **b** HCT116 *Bax*^*−/−*^ cells were transfected with pcDNA3.1-Bax (**a**) or pcDNA3.1-BAK (**b**) and examined by western blotting for the expression of the proteins. **c** After HCT116 or Bax-overexpressing or BAK-overexpressing HCT116 *Bax*^*−/−*^ cells treated with sanguinarine for 24 h, apoptosis was detected. (TIF 1013 kb)

Additional file 7: **Figure S7.** Comparison of the mitochondrial membrane potential in HCT116 WT and HCT116 *Bax*^*−/−*^ treated with sanguinarine. Cells were treated with the various concentrations of Sanguinarine and stained with JC-1. Mean JC-1 fluorescence intensity was evalued by a flow cytometer. (TIF 6622 kb)

Additional file 8: **Figure S8.** HCT116 WT and HCT116 *Bax*^*−/−*^ cells were treated with sanguinarine for 12 h. RNA was extracted and qRT-PCR were performed to analyze MELK mRNA relative level. ^#^ *p* < 0.01 indicates significant difference. (TIF 2220 kb)

Additional file 9: **Figure S9.** SW480 cells were treated with sanguinarine for indicated time. The experession of Caspase 8 protein was examined using western blotting. (TIF 614 kb)

## Abbreviations

CRC: Colorectal cancer
MELK: Maternal embryonic leucine zipper kinase
MOMP: Mitochondrial outer membrane permeabilization
STRAP: Serine-threonine kinase receptor-associated protein
