# Supplementary figures and images for: Sanguinarine triggers intrinsic apoptosis to suppress colorectal cancer growth through disassociation between STRAP and MELK
Source: BMC Cancer. 2018 May 21;18:578. doi: 10.1186/s12885-018-4463-x (PMC5963096; doi:10.1186/s12885-018-4463-x)

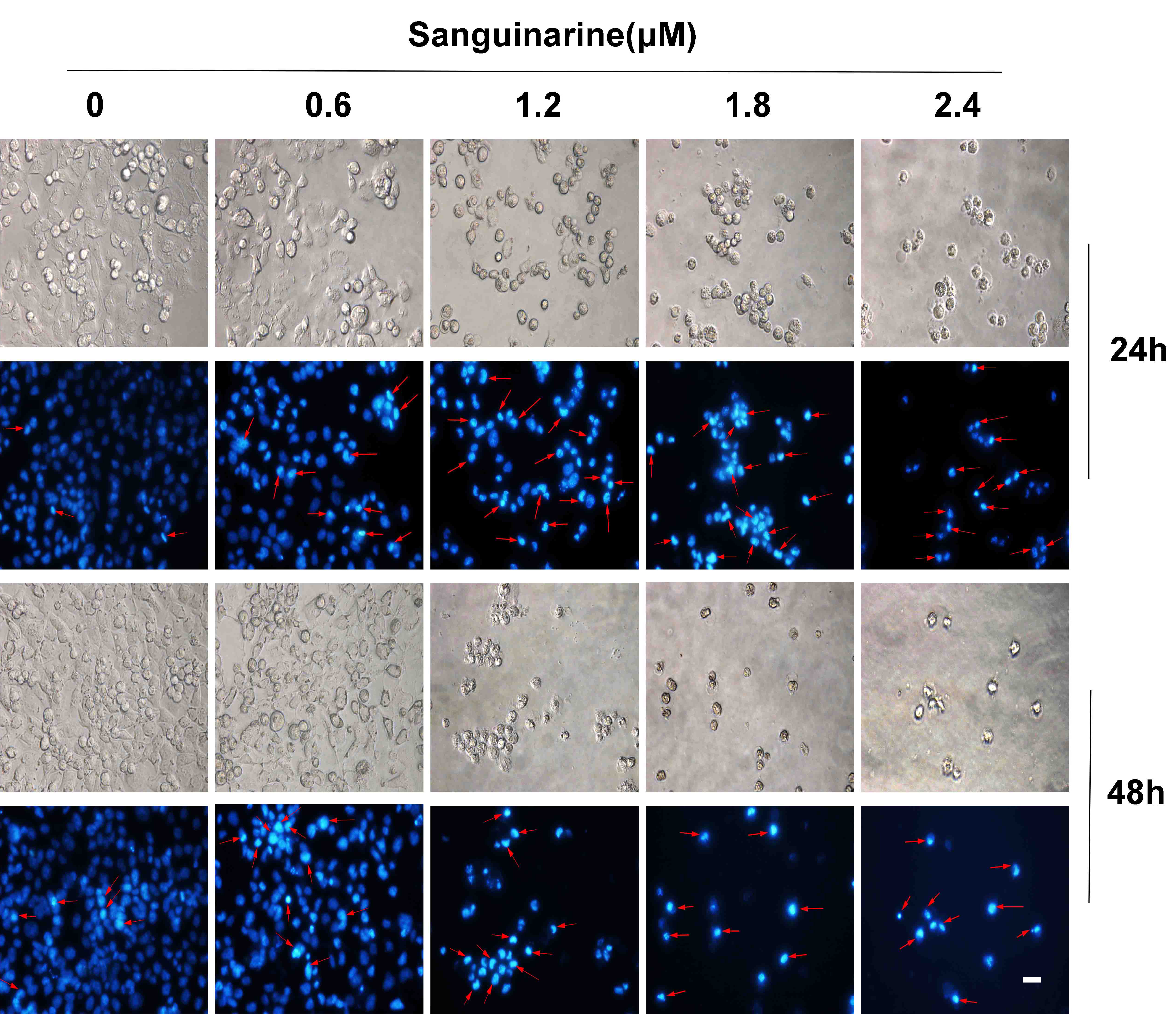

Supplement: Supplementary file 1 — Figure S1. Apoptosis was detected in SW480 cells treated with sanguinarine. Cells were treated with the indicated concentrations of sanguinarine for 24 h and 48 h and then stained by DNA-specific Hoechst 33,342 dye. Imagines were observed by inverted fluorescence microscope (400×). Scale bar is 50 μm. (TIF 16868 kb) [file 12885_2018_4463_MOESM1_ESM.tif]

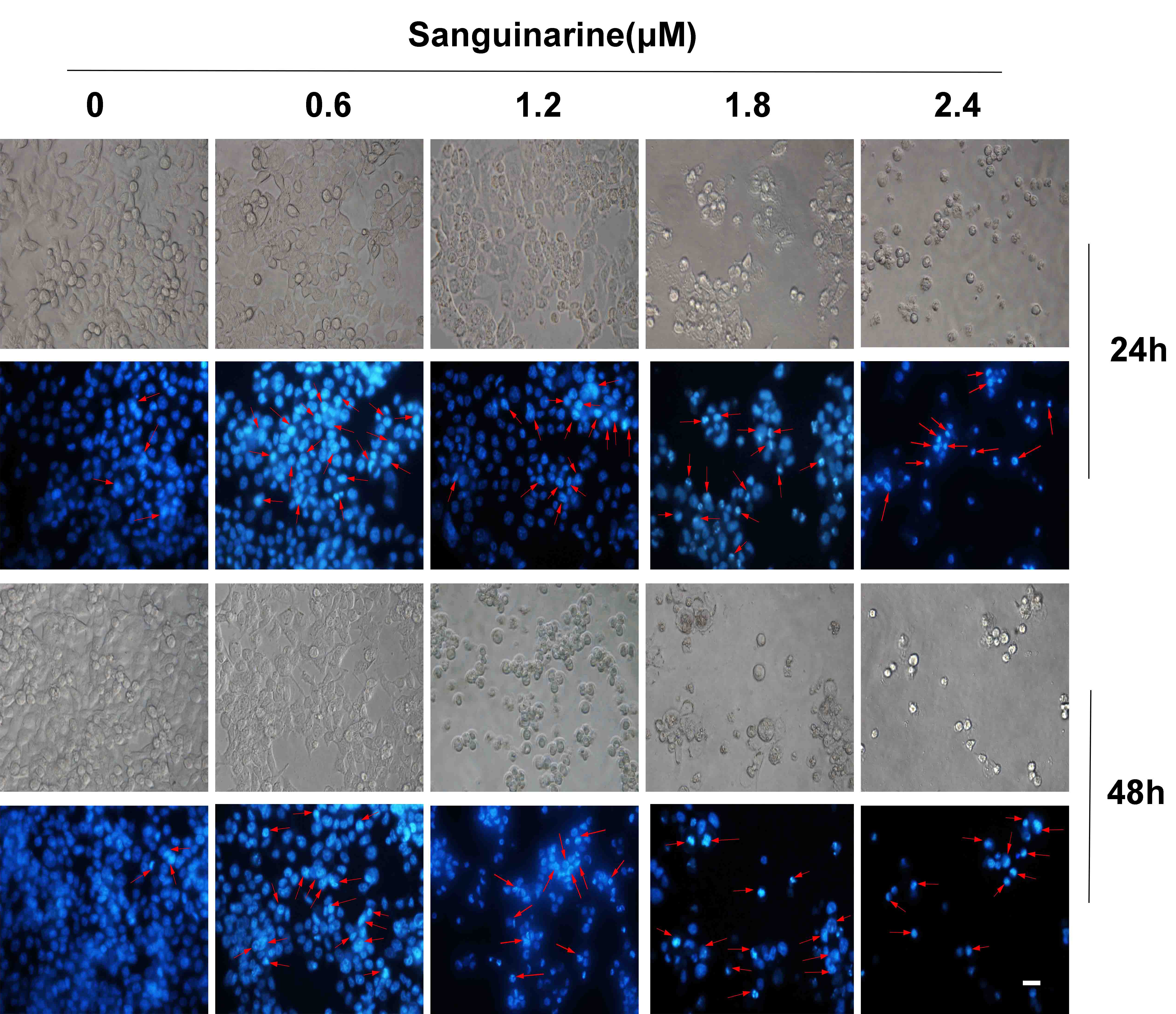

Supplement: Supplementary file 2 — Figure S2. Apoptosis was detected in HCT116 cells treated with sanguinarine. Cells were treated with the indicated concentrations of sanguinarine for 24 h and 48 h and then stained by DNA-specific Hoechst 33,342 dye. Imagines were observed by inverted fluorescence microscope (400×). Scale bar is 50 μm. (TIF 16794 kb) [file 12885_2018_4463_MOESM2_ESM.tif]

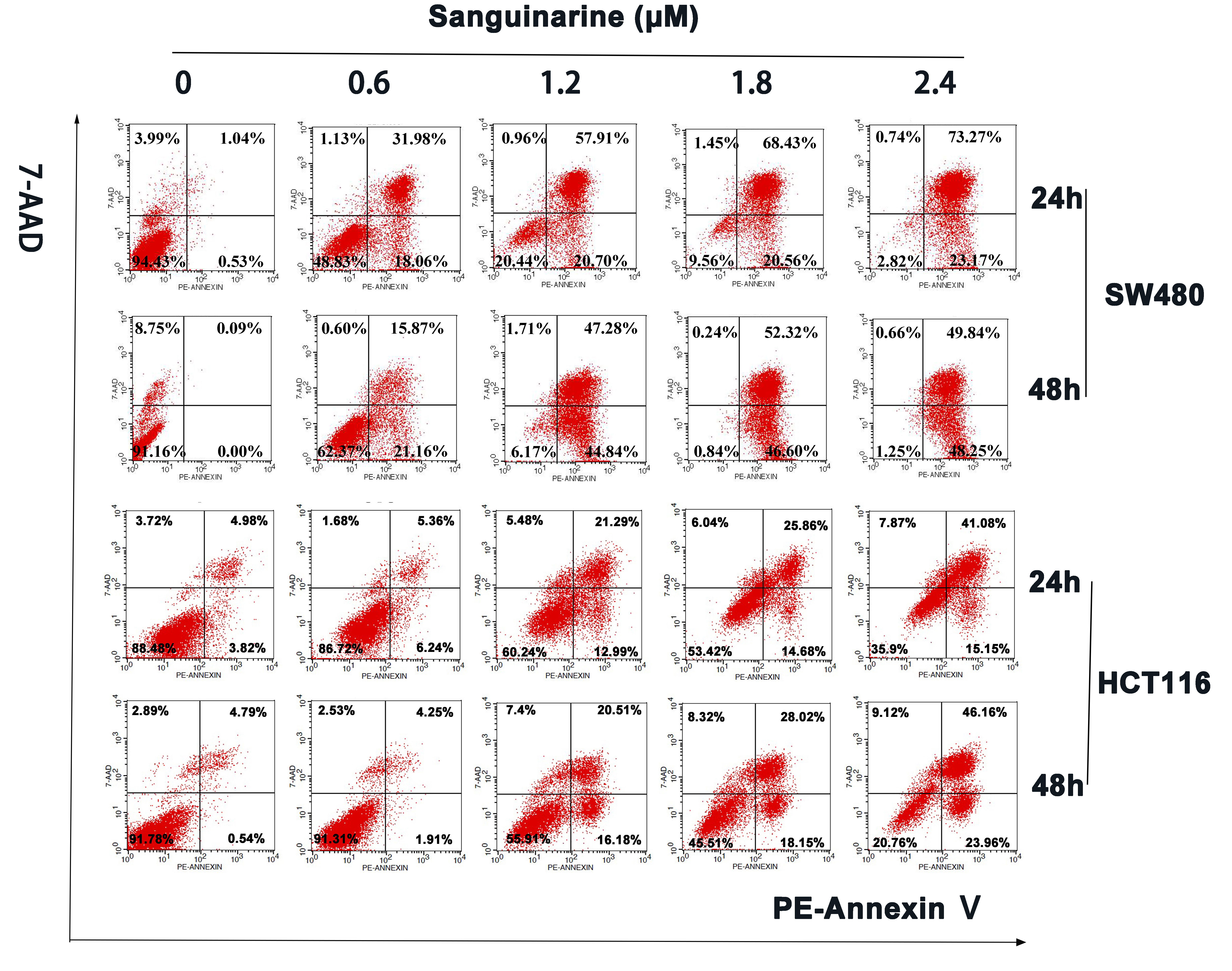

Supplement: Supplementary file 3 — Figure S3. Apoptotic cells were significantly increased by sanguinarine treatment. Indicated CRC cell lines were treated with the various concentrations of sanguinarine. To detect the degree of apoptosis, cells were analyzed by flow cytometry after PE-Annexin V and 7-AAD staining. (TIF 15845 kb) [file 12885_2018_4463_MOESM3_ESM.tif]

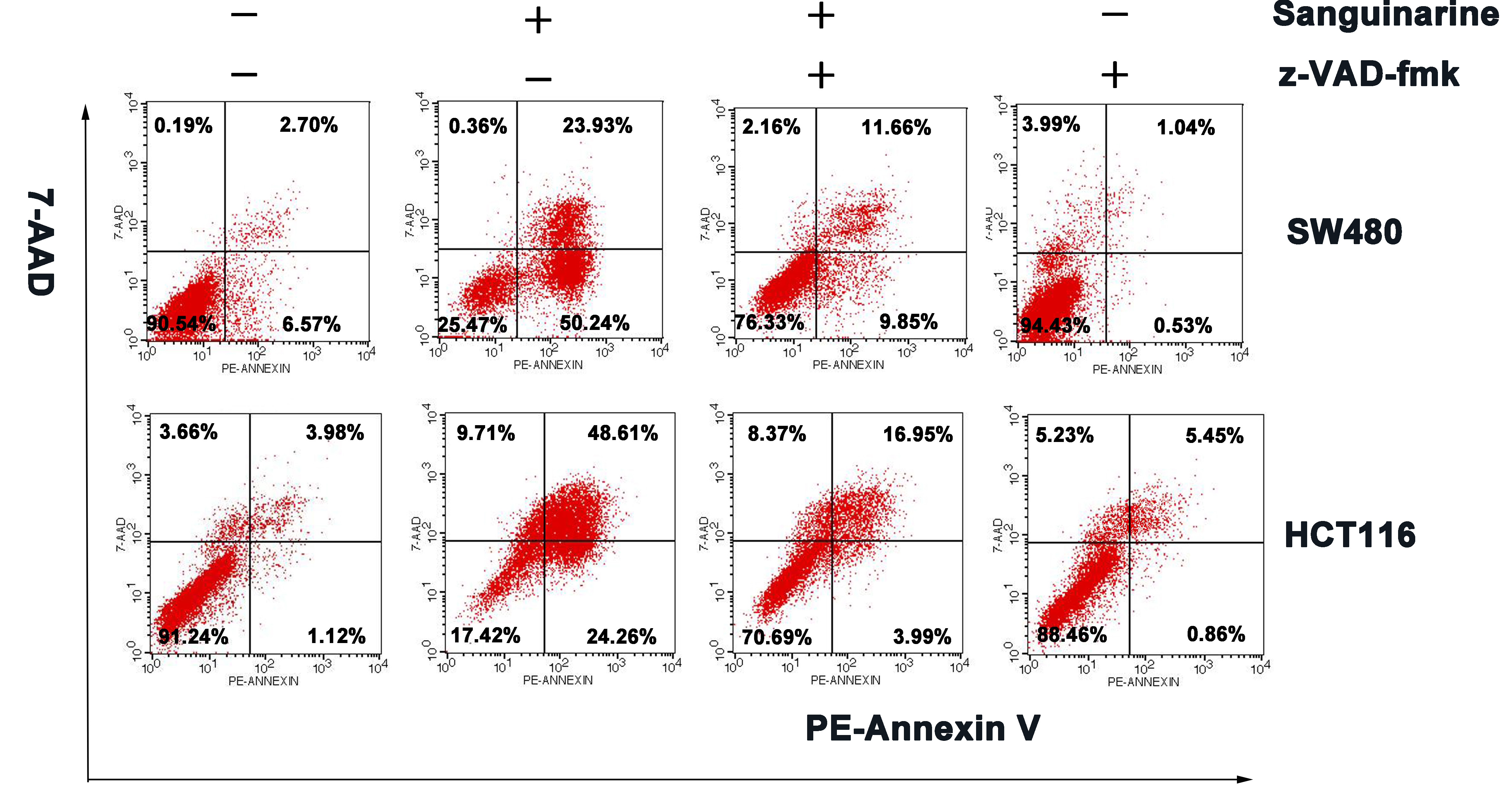

Supplement: Supplementary file 4 — Figure S4. Z-VAD-fmk inhibited sanguinarine-induced apoptosis in CRC cells. After treatment with the combination of sanguinarine and Z-VAD-fmk (50 μM), cells were analyzed using flow cytometry. (TIF 5542 kb) [file 12885_2018_4463_MOESM4_ESM.tif]

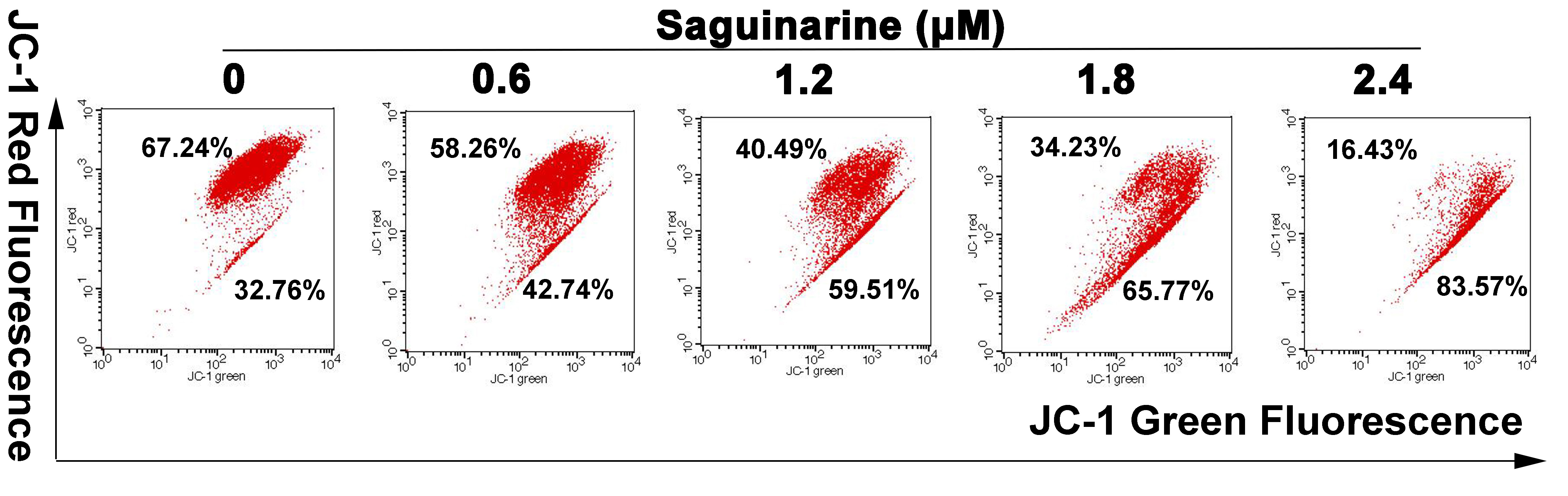

Supplement: Supplementary file 5 — Figure S5. Change of the mitochondrial membrane potential (MMP) in SW480 cells by sanguinarine. Cells were treated with the various concentrations of sanguinarine and stained with JC-1. Mean JC-1 fluorescence intensity was evalued by a flow cytometer. (TIF 2721 kb) [file 12885_2018_4463_MOESM5_ESM.tif]

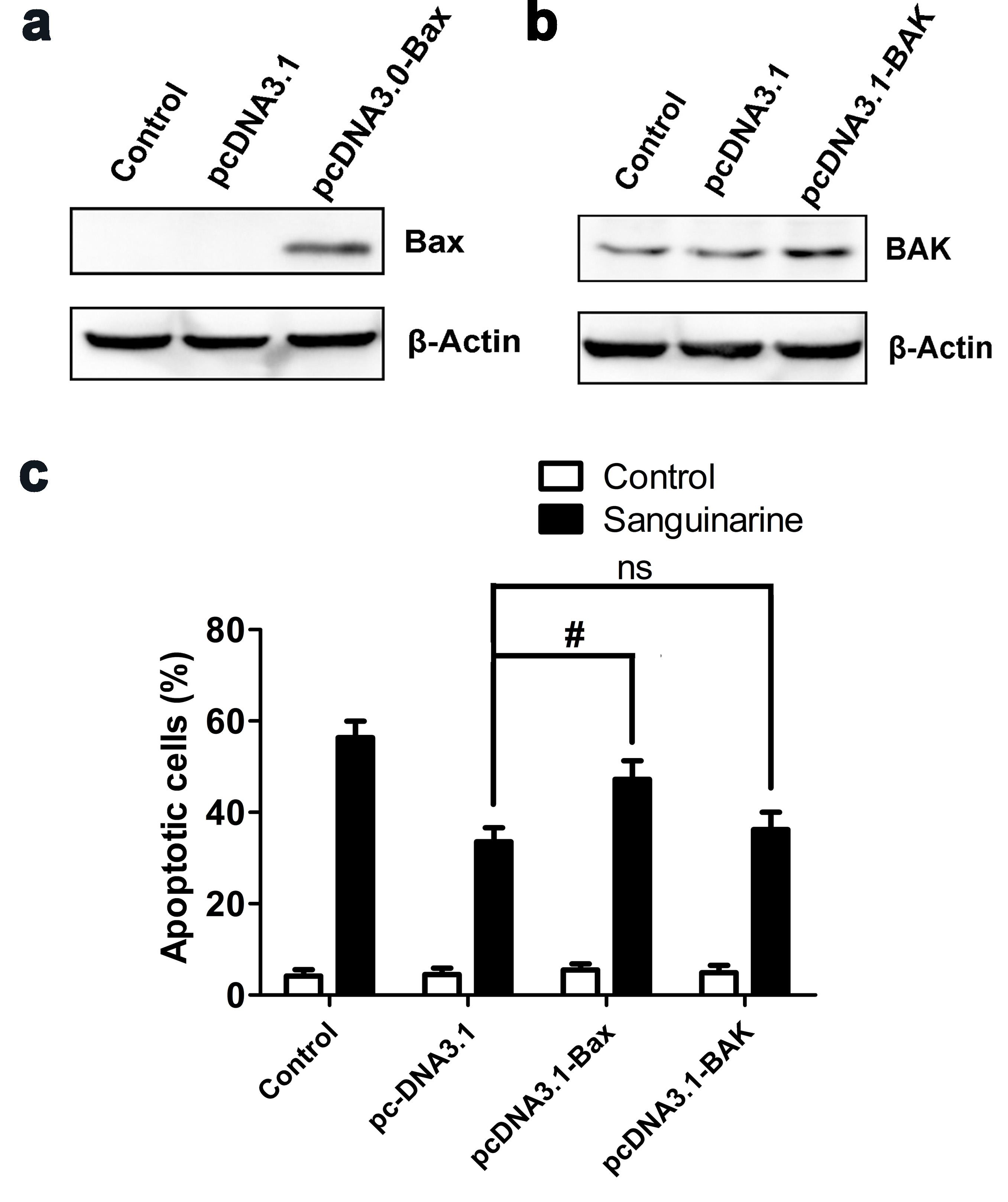

Supplement: Supplementary file 6 — Figure S6. Detection of Apoptosis in Bax-overexpressing or BAK-overexpressing HCT116 Bax−/− cells treated with sanguinarine. a and b HCT116 Bax−/− cells were transfected with pcDNA3.1-Bax (a) or pcDNA3.1-BAK (b) and examined by western blotting for the expression of the proteins. c After HCT116 or Bax-overexpressing or BAK-overexpressing HCT116 Bax−/− cells treated with sanguinarine for 24 h, apoptosis was detected. (TIF 1013 kb) [file 12885_2018_4463_MOESM6_ESM.tif]

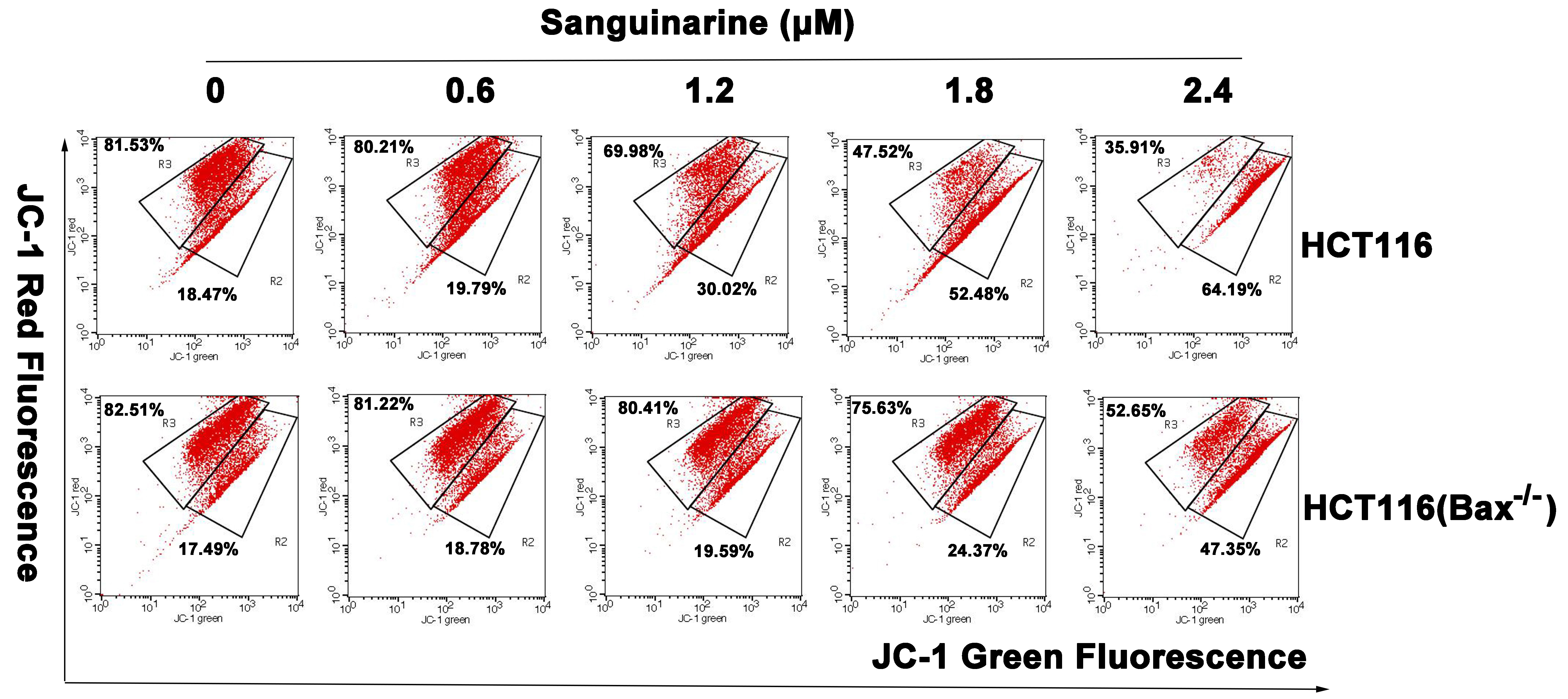

Supplement: Supplementary file 7 — Figure S7. Comparison of the mitochondrial membrane potential in HCT116 WT and HCT116 Bax−/− treated with sanguinarine. Cells were treated with the various concentrations of Sanguinarine and stained with JC-1. Mean JC-1 fluorescence intensity was evalued by a flow cytometer. (TIF 6622 kb) [file 12885_2018_4463_MOESM7_ESM.tif]

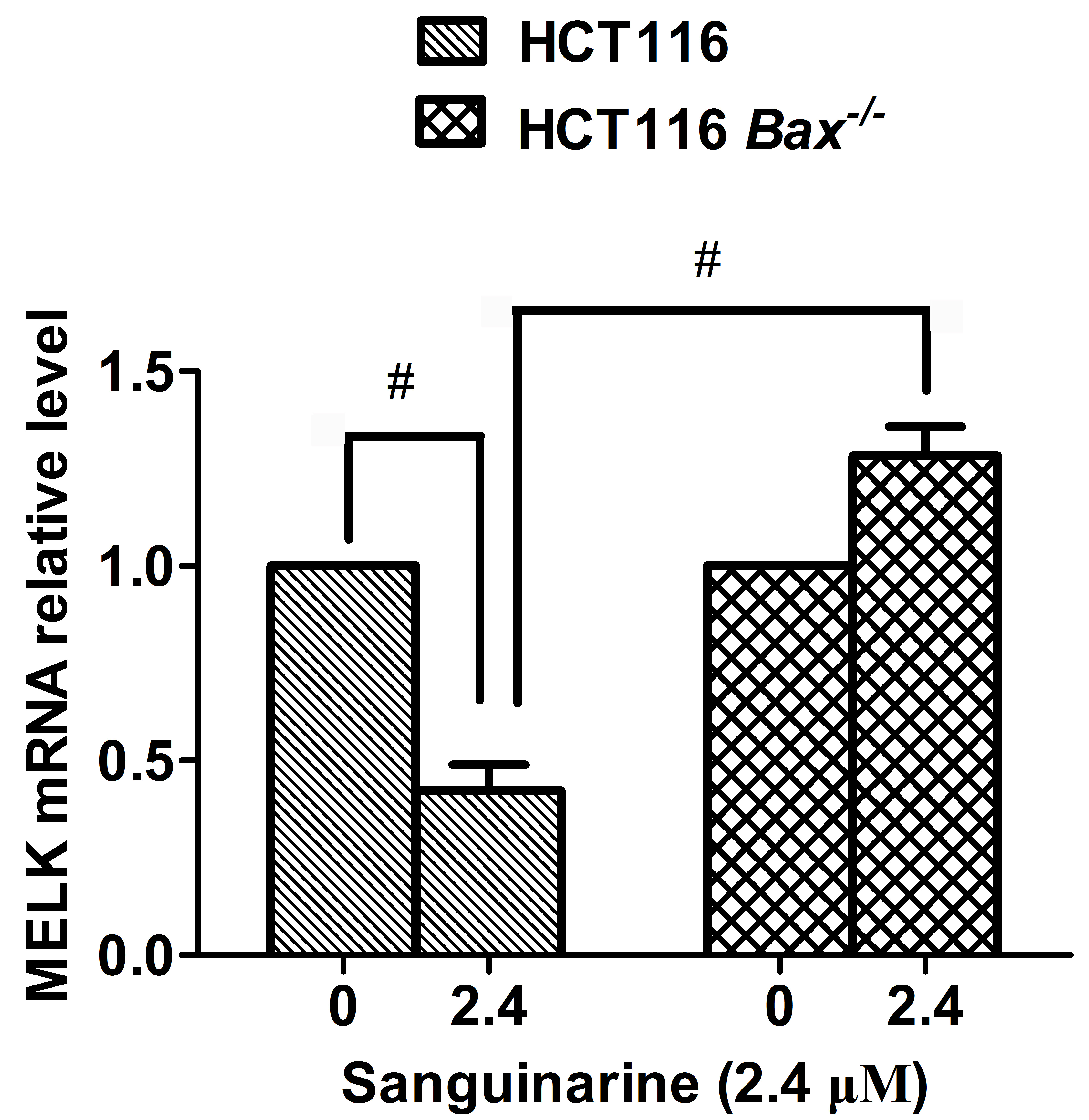

Supplement: Supplementary file 8 — Figure S8. HCT116 WT and HCT116 Bax−/− cells were treated with sanguinarine for 12 h. RNA was extracted and qRT-PCR were performed to analyze MELK mRNA relative level. # p < 0.01 indicates significant difference. (TIF 2220 kb) [file 12885_2018_4463_MOESM8_ESM.tif]

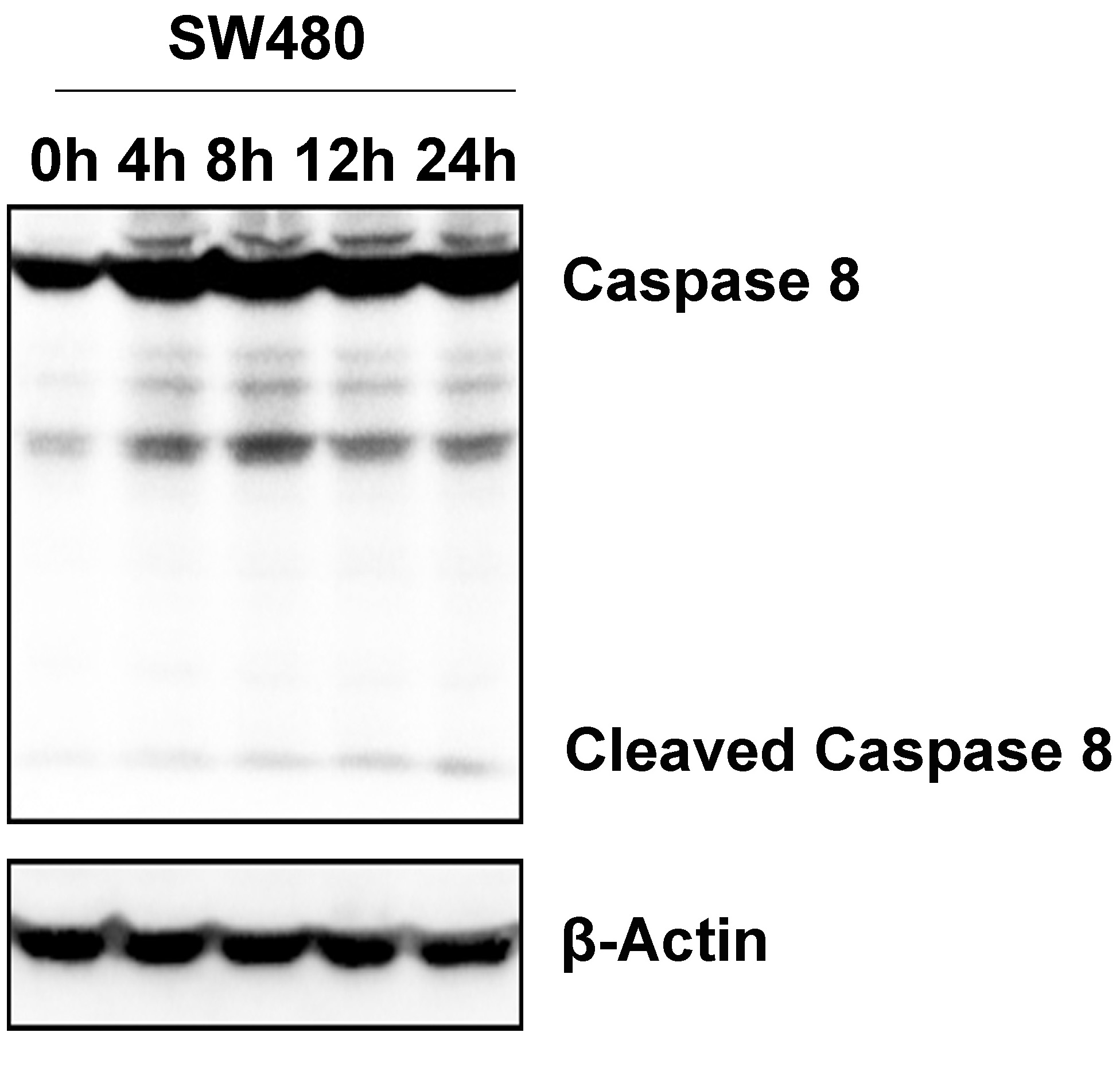

Supplement: Supplementary file 9 — Figure S9. SW480 cells were treated with sanguinarine for indicated time. The experession of Caspase 8 protein was examined using western blotting. (TIF 614 kb) [file 12885_2018_4463_MOESM9_ESM.tif]
